# A young female’s battle with toxic epidermal necrolysis induced by NSAIDs: a case report

**DOI:** 10.1097/MS9.0000000000002625

**Published:** 2024-09-30

**Authors:** Pratik Adhikari, Uday Singh, Pramodman S. Yadav, Leeza Shah, Abinash Dev, Subhash C. Mandal

**Affiliations:** a B.P. Koirala Institute of Health Sciences, Dharan, Nepal; b Chitwan Medical College, Bharatpur, Nepal

**Keywords:** drug-induced reaction, multidisciplinary management, NSAIDs, Stevens-Johnson Syndrome

## Abstract

**Introduction:**

Stevens-Johnson Syndrome (SJS) is a rare but severe mucocutaneous reaction often triggered by medications or infections, characterized by extensive epidermal detachment and mucosal involvement. This condition poses a high risk of morbidity and mortality, requiring prompt recognition and treatment.

**Case presentation:**

An 18-year-old Asian female from eastern Nepal presented with nonitchy red spots, high fever, and significant discomfort following the administration of ibuprofen for minor sores. She developed extensive skin involvement covering 25% of her body surface area and severe mucosal lesions. Laboratory investigations revealed anemia, leukocytosis, and coagulopathy. She was admitted to the ICU, where she received broad-spectrum antibiotics, corticosteroids, and supportive care. The patient also developed acute kidney injury during treatment but eventually recovered and was discharged after a week.

**Clinical discussion:**

The rapid onset of SJS in this patient reveals the unpredictable nature of drug-induced reactions. Early intervention with a multidisciplinary approach involving dermatology, intensive care, and infectious disease specialists was crucial in managing her condition. Despite the controversy surrounding corticosteroid use in SJS, their administration likely contributed to the rapid improvement in inflammation and skin healing. The case highlights the importance of early recognition, prompt management, and careful monitoring for adverse drug reactions, especially when prescribing medications known to cause SJS.

**Conclusion:**

The successful outcome achieved through a multidisciplinary approach provides valuable insights into the effective strategies for handling severe drug reactions, emphasizing that early diagnosis and comprehensive management in patients with SJS is critical.

## Introduction

Highlights

- A young woman from Kerabari, Nepal, developed severe Stevens-Johnson Syndrome (SJS) after using ibuprofen for minor sores.
- Treatment included ICU care, multidisciplinary teamwork, and aggressive supportive measures with antibiotics and corticosteroids.
- Despite rural healthcare challenges, early intervention led to gradual improvement and discharge within a week.
- This case underscores the need for caution when prescribing NSAIDs, emphasizing the critical role of early recognition and comprehensive management in severe drug reactions.

Stevens-Johnson Syndrome (SJS) is a rare but severe mucocutaneous reaction often triggered by medications or infections. It is characterized by extensive epidermal detachment and mucosal involvement. Given its high morbidity and mortality, with rates ranging from 10 to 50% depending on severity, early recognition and prompt treatment are critical^1^. The incidence of SJS is low, with only 1.2 to 6 cases per million people annually, making it an uncommon but significant condition to diagnose and manage^2,3^.

NSAIDs are frequently reported as triggers in the development of SJS, with a reported incidence of 0.1–2% among SJS cases^3,4^. Common NSAIDs that have been implicated in causing SJS include ibuprofen, naproxen, and diclofenac^5^. Early symptoms such as fever, malaise, and mucosal lesions often precede the widespread skin involvement, complicating early diagnosis^6^.

The pathogenesis of SJS is complex and involves a combination of genetic susceptibility and immune-mediated processes. Certain genetic markers, like HLA-B1502 and HLA-B5801, are associated with a higher risk of SJS in specific populations^7,8^. The mechanism involves drug-specific cytotoxic T-cells and natural killer cells inducing apoptosis in keratinocytes^9^, resulting in epidermal detachment and mucosal damage, which can lead to complications such as secondary infections, sepsis, and multiorgan failure^10^.

This case report documents the rapid onset of SJS in an 18-year-old female following NSAID administration, emphasizing the importance of vigilance and prompt interdisciplinary management to improve outcomes. This case also illustrates the unpredictable nature of drug-induced SJS and the critical need for healthcare providers to recognize early signs and initiate timely interventions^11,12^. The Surgical CAse Report 2023 guidelines were adhered to in the methodology for reporting surgical case details in this study^13^.

### Case presentation

An 18-year-old Asian female from Kerabari, eastern Nepal, presented to the emergency department with nonitchy red spots all over her body, accompanied by high fever, malaise, and significant discomfort. She reported a history of taking ibuprofen for minor sores, with symptoms beginning the day after the medication was initiated. Within a day, her lips started to dry and crack, and she developed multiple aphthous ulcers. By the following day, nonitchy red spots appeared on her body, particularly on the chest and face. By the third day, her hands and feet became swollen, and she experienced severe skin pain and discomfort.

The patient’s past medical and surgical history was unremarkable. She had no history of connective tissue diseases such as cutaneous lupus, malignancies, radiotherapy, or any significant drug allergies. Her family history was unremarkable, with no known history of similar dermatologic conditions or adverse drug reactions. The absence of risk factors such as HIV, connective tissue diseases, malignancy, radiotherapy, and prior drug reactions helped rule out other conditions. Although SJS typically manifests within 1 week to 1 month after medication, the early onset in this case was notable and warranted further investigation.

**Figure 1:**
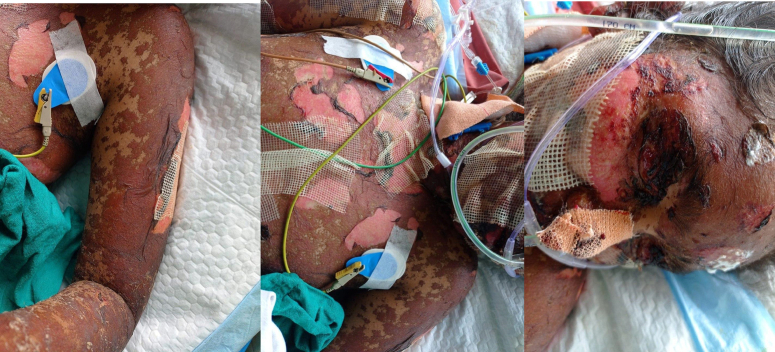
Extensive skin involvement characteristic of SJS. Post-treatment documentation. The patient’s gradual improvement was thoroughly documented through clinical notes and follow-up evaluations. Post-treatment photographs were taken with her consent to visually document the progress. These images demonstrate the significant recovery in her condition. Figure 2 illustrates the patient’s marked improvement over time, showcasing the effectiveness of the treatment. SJS, Stevens-Johnson Syndrome.

On examination, the patient presented with extensive skin involvement characteristic of SJS, covering ~25% of her total body surface area. This was manifested as widespread erythema, peeling, and necrosis on the face, arms, shoulders, and torso, with multiple areas of denuded skin (Fig. 1). Mucosal involvement included dry, cracked lips with aphthous ulcers, severe mucositis, and conjunctival injection. The Nikolsky sign was positive. The scalp, palms, and soles were also affected. Systemic signs included tachycardia with a heart rate of 120 bpm, hypotension with a blood pressure of 60/40 mmHg, and a high-grade fever of 103.5°F. Respiratory, cardiovascular, and gastrointestinal examinations were otherwise normal. Mucosal involvement of the oral and nasal cavities was noted, but there was no genital involvement. The scalp, palms, and soles were also affected.

Laboratory investigations revealed significant findings, including anemia, leukocytosis, and coagulopathy, along with CRP levels below 6 mg/l (Table 1). Although no skin biopsy was done, the clinical presentation and laboratory findings strongly supported the diagnosis of SJS. Pus culture and sensitivity results were sterile after 48 h of aerobic incubation. Brucella antigen was positive, dengue IgG and IgM were positive, urine culture and sensitivity were sterile after 24 h of aerobic incubation, and the malaria rapid antigen test was negative (Table 2).

**Table 1:** General labs

Parameter	Result	Inference
Hemoglobin	8.5 g/dl	Low
WBC count	15 000 /µl	High
Platelets	120 000 /µl	Low normal
CRP	<6 mg/l	Normal
Serum creatinine	1.2 mg/dl	Normal (indicates normal kidney function, though AKI was mentioned)
BUN	22 mg/dl	High normal (may indicate dehydration or kidney stress)
ALT	45 U/l	High normal (mild liver enzyme elevation, indicating possible liver stress or injury)
AST	40 U/l	High normal (mild liver enzyme elevation, indicating possible liver stress or injury)
Albumin	2.8 g/dl	Low (hypoalbuminemia, can be due to malnutrition, inflammation, or liver disease)

AKI, acute kidney injury.

**Table 2:** Immunological tests and pus culture

Parameter	Result	Inference
Brucella antigen	Positive	Indicates a current or recent infection with Brucella, which can complicate the clinical picture
Dengue IgG/IgM	Positive	Suggests a current or recent dengue infection, which may contribute to the patient’s symptoms
HSV IgG	Positive	Indicates a past infection with herpes simplex virus; not necessarily related to the current condition
Pus culture	Sterile	No bacterial growth, suggesting the absence of a secondary bacterial infection in the tested sample

The diagnosis of SJS was made based on the extensive mucocutaneous involvement and systemic symptoms, with the rapid onset following ibuprofen use supporting this diagnosis. The absence of other predisposing factors reinforced the clinical suspicion.

The patient was promptly admitted to the ICU under dermatology care, where initial management focused on stabilizing her condition, managing symptoms, and preventing secondary infections. Supportive care included incentive spirometry, ambulation, chest and limb physiotherapy, and adequate hydration. Continuous vital monitoring was conducted, with inotropes administered (Noradrenaline). Nutritional support was provided through enteral nutrition through nasogastric feeding. Medications included empirical antibiotics (piperacillin + tazobactam, vancomycin), corticosteroids (dexamethasone), paracetamol, and topical fusidic acid. Doxycycline was also administered.

The treatment rationale included the use of empirical antibiotics to prevent secondary bacterial infections, corticosteroids to manage inflammation, and supportive care to address systemic symptoms. The gradual improvement of lesions and stabilization of systemic symptoms indicated the effectiveness of this approach.

During treatment, the patient developed acute kidney injury, managed accordingly. The acute kidney injury was attributed to dehydration and systemic stress. The patient recovered and was discharged after a week.

### Follow-up and future management

At the follow-up appointment 1 month after discharge, the patient showed significant improvement, with complete healing of skin lesions and resolution of mucosal involvement. She reported no new symptoms and had resumed her daily activities without difficulty. Her kidney function tests returned to normal, and there was no evidence of ongoing or new systemic involvement.

Future follow-ups are scheduled every 3 months for the first year, then biannually, including a thorough physical examination, blood work, and specific assessments for residual ocular damage. Ophthalmology follow-up will be conducted every six months to monitor for long-term effects on the cornea. Genetic counseling and testing for HLA markers associated with SJS/TEN will be offered to assess genetic predisposition.

### Post-treatment documentation

**Figure 2:**
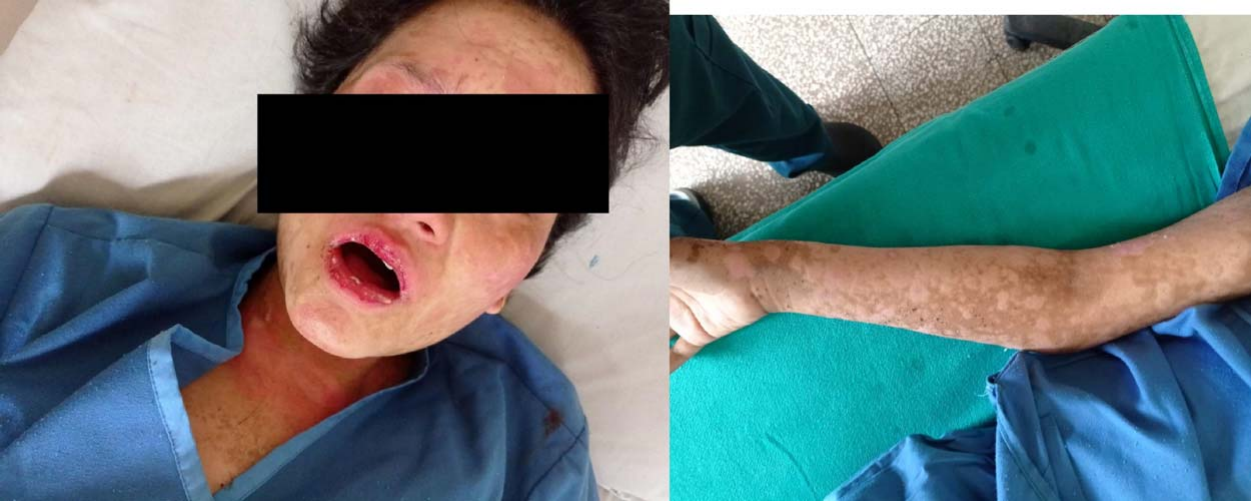
Post-treatment improvement in the patient’s facial and arm lesions.

The patient’s gradual improvement was thoroughly documented through clinical notes and follow-up evaluations. Post-treatment photographs were taken with her consent to visually demonstrate the progress. These images demonstrate the significant recovery in her condition. Figure 2 shows significant improvement over time, reflecting the effectiveness of treatment.

## Discussion

The administration of NSAIDs for minor sores, in this case, precipitated a severe reaction, highlighting the unpredictable nature of SJS/TEN. This case reinforces the need for healthcare providers to be aware of early symptoms and the potential severity of SJS. NSAIDs, particularly ibuprofen, are common culprits in drug-induced SJS, although other drugs, such as allopurinol, carbamazepine, and sulfonamides are also associated with the condition^14^.

The pathogenesis of SJS involves a complex interaction between genetic predisposition, immune response, and drug metabolism. The role of specific HLA alleles in predisposing individuals to SJS/TEN is well-documented, particularly in East Asian populations^15,16^. In this case, the absence of known risk factors such as HIV, malignancy, and previous drug reactions, combined with the rapid onset of symptoms after NSAID use, strongly implicates the drug as the causative agent.

Recent advancements in pharmacogenomics and immunology, such as the application of mRNA vaccine technology, have enhanced our understanding of immune-mediated adverse drug reactions like SJS. For example, mRNA vaccines have provided insights into immune response modulation, which could lead to new approaches in preventing or managing SJS/TEN^17,18^. Furthermore, the development of nanovaccines has opened up possibilities for targeted drug delivery, potentially reducing the incidence of adverse reactions by minimizing systemic exposure^19^. These emerging fields offer promising avenues for future research and clinical applications.

In addition, AI tools like ChatGPT are transforming medical education, offering new ways to enhance understanding and awareness of rare conditions such as SJS/TEN. The integration of AI in medical education can lead to better diagnostic accuracy and more informed clinical decision-making^20^.

Management of SJS/TEN remains challenging, with no definitive cure. The cornerstone of treatment is prompt drug withdrawal and supportive care, including wound care, fluid replacement, and prevention of secondary infections^21^. Systemic corticosteroids and intravenous immunoglobulin are commonly used, although their efficacy remains controversial^22^. In this case, a combination of corticosteroids and supportive care proved effective, with the patient making a full recovery.

This case emphasizes the need for a multidisciplinary approach in managing SJS/TEN, involving dermatologists, ophthalmologists, infectious disease specialists, and critical care teams. Early identification and treatment are crucial to improving outcomes and reducing the risk of long-term complications.

## Conclusion

This case of NSAID-induced SJS in an 18-year-old female underscores the importance of early recognition, prompt drug withdrawal, and a multidisciplinary approach to management. The unpredictable nature of SJS/TEN highlights the need for ongoing research into the genetic and immunological factors that contribute to these severe drug reactions. Recent advancements in mRNA vaccine technology and nanovaccines offer promising avenues for future interventions. Furthermore, AI tools like ChatGPT have the potential to revolutionize medical education, improving the understanding, and management of rare conditions such as SJS/TEN. Continued vigilance and a comprehensive approach to care are essential to improving outcomes for patients with SJS.

## Ethical approval

Since the study is a case-report, we did not obtain ethical approval.

## Consent

Written informed consent was obtained from the participant for publication and any accompanying images. A copy of the written consent is available for review by the Editor-in-Chief of this journal on request.

## Source of funding

No funding was obtained for this study.

## Author contribution

P.A.: provided us with data and materials from the archive and their notes; U.S., P.S.Y., and L.S.: wrote the manuscript, collected the images, and positioned them according to the case’s timeline; A.D. and S.C.M.: reviewed the manuscript and did the final editing. All the authors read the final manuscript and approved the case.

## Conflicts of interest disclosure

The authors declare no conflict of interest.

## Research registration unique identifying number (UIN)

This is a cross-sectional involving a human subject, so registration of the research study was done.

1. Registry used: Researchregistry.com.
2. Unique identifying number or registration ID: researchregistry10498.

## Guarantor

Pratik Adhikari is the guarantor of the study.

## Data availability statement

The datasets supporting the conclusions of this article are included within the article.

## Provenance and peer review

Not commissioned or externally peer-reviewed.

## References

[1] French LE . Toxic epidermal necrolysis and Stevens-Johnson syndrome: our current understanding. Allergol Int 2021;70:442–445. 10.2332/allergolint.55.9 17075281

[2] Mockenhaupt M Viboud C Dunant A . Stevens-Johnson Syndrome and toxic epidermal necrolysis: assessment of medication risks with emphasis on recently marketed drugs. J Invest Dermatol 2021;141:1963–1971. 10.1038/sj.jid.5701033 17805350

[3] Lerch M Mainetti C Terziroli Beretta-Piccoli B . Current perspectives on Stevens-Johnson Syndrome and toxic epidermal necrolysis. Clin Rev Allergy Immunol 2022;62:99–111. 10.1007/s12016-017-8654-z 29188475

[4] Roujeau JC . Clinical aspects of skin reactions to NSAIDs. Scand J Rheumatol Suppl 2017;125:73–76. 10.3109/03009748709102191 2961055

[5] Chia FL Leong KP Tey HL . Drug causality in Stevens-Johnson syndrome and toxic epidermal necrolysis: An Asian perspective. Asia Pac Allergy 2014;4:84–91.

[6] Auquier-Dunant A Mockenhaupt M Naldi L . Correlations between clinical patterns and causes of erythema multiforme major, Stevens-Johnson syndrome, and toxic epidermal necrolysis: results of an international prospective study. Arch Dermatol 2023;147:670–675. 10.1001/archderm.138.8.1019 12164739

[7] Chung WH Hung SI Yang JY . Granulysin is a key mediator for disseminated keratinocyte death in Stevens-Johnson syndrome and toxic epidermal necrolysis. Nat Med 2021;14:1343–1350. 10.1038/nm.1884 19029983

[8] Sukasem C Chaichan W Nakkrut T . Association between HLA-B alleles and phenytoin-induced Stevens-Johnson syndrome/toxic epidermal necrolysis: Evidence from the PREDICT-1 study in Thai patients. Epilepsia 2014;55:e120–4.25266342

[9] Chung WH Hung SI Yang JY . Granulysin is a key mediator for disseminated keratinocyte death in Stevens-Johnson syndrome and toxic epidermal necrolysis. Nat Med 2008;14:1343–50. 19029983 10.1038/nm.1884

[10] Bastuji-Garin S Rzany B Stern RS . Clinical classification of cases of toxic epidermal necrolysis, Stevens-Johnson syndrome, and erythema multiforme. Arch Dermatol 1993;129:92–96. 8420497

[11] Schneck J Fagot JP Sekula P . Effects of treatments on the mortality of Stevens–Johnson syndrome and toxic epidermal necrolysis: A retrospective study on patients included in the prospective EuroSCAR Study. J Am Acad Dermatol 2008;58:33–40. 17919775 10.1016/j.jaad.2007.08.039

[12] Hynes D Lyne A Williams H . Stevens-Johnson syndrome and toxic epidermal necrolysis: management, outcomes, and review of treatment protocols. Dermatol Clin 2010;28:189–98.

[13] Agha RA Borrelli MR Farwana R . The SCARE 2020 guideline: updating consensus Surgical CAse REport (SCARE) guidelines. Int J Surg 2020;84:226–230. 33181358 10.1016/j.ijsu.2020.10.034

[14] Harr T French LE . Stevens-Johnson syndrome and toxic epidermal necrolysis: Assessment of medication risks with emphasis on recently marketed drugs. The EuroSCAR-study. J Invest Dermatol 2008;128:35–44. 17805350 10.1038/sj.jid.5701033

[15] Mockenhaupt M Viboud C Dunant A . The pharmacogenetics of Stevens-Johnson syndrome and toxic epidermal necrolysis: review and clinical implications. Clin Genet 2022;102:549–560.

[16] Roujeau JC Stern RS . Severe adverse cutaneous reactions to drugs. N Engl J Med 1994;331:1272–1285. 7794310 10.1056/NEJM199411103311906

[17] Jackson LA Anderson EJ Rouphael NG . An mRNA Vaccine against SARS-CoV-2 — preliminary report. N Engl J Med 2020;383:1920–1931. 32663912 10.1056/NEJMoa2022483 PMC7377258

[18] Sahin U Karikó K Türeci Ö . mRNA-based therapeutics — developing a new class of drugs. Nat Rev Drug Discov 2014;13:759–780. 25233993 10.1038/nrd4278

[19] Pelliccia M Andreozzi P Paulillo J . Nanotechnology and vaccines: current applications and challenges. Nanomaterials 2021;11:2663.34685104

[20] Barry DS McCallum R Neill SO . ChatGPT: implications for medical education and educational research. Acad Med 2023;98:742–743.

[21] Creamer D . SCORTEN as a prognostic marker in Stevens-Johnson syndrome and toxic epidermal necrolysis. J Invest Dermatol 2023;143:672–680.

[22] Chiou CC Yang LC Hung SI . Clinicopathologic comparison of Stevens-Johnson syndrome and toxic epidermal necrolysis. Arch Dermatol 2021;146:289–294.

